# Effects of volume resuscitation on the microcirculation in animal models of lipopolysaccharide sepsis: a systematic review

**DOI:** 10.1186/s40635-016-0112-3

**Published:** 2016-11-21

**Authors:** Nchafatso G. Obonyo, Jonathon P. Fanning, Angela S. Y. Ng, Leticia P. Pimenta, Kiran Shekar, David G. Platts, Kathryn Maitland, John F. Fraser

**Affiliations:** 1 Critical Care Research Group, The Prince Charles Hospital, Brisbane, Queensland Australia; 2 Kenya Medical Research Institute-Wellcome Trust Research Programme, Kilifi, Kenya; 3 School of Medicine, University of Queensland, Brisbane, Queensland Australia; 4 Department of Paediatrics, Faculty of Medicine, Imperial College London, London, UK

**Keywords:** Lipopolysaccharide, Sepsis, Septic shock, Fluid resuscitation, Microcirculation, Intravital fluorescence microscopy, Oxygen extraction, Laser Doppler flowmetry, Sidestream dark-field videomicroscopy, Laser speckle imaging

## Abstract

**Background:**

Recent research has identified an increased rate of mortality associated with fluid bolus therapy for severe sepsis and septic shock, but the mechanisms are still not well understood. Fluid resuscitation therapy administered for sepsis and septic shock targets restoration of the macro-circulation, but the pathogenesis of sepsis is complex and includes microcirculatory dysfunction.

**Objective:**

The objective of the study is to systematically review data comparing the effects of different types of fluid resuscitation on the microcirculation in clinically relevant animal models of lipopolysaccharide-induced sepsis.

**Methods:**

A structured search of PubMed/MEDLINE and EMBASE for relevant publications from 1 January 1990 to 31 December 2015 was performed, in accordance with PRISMA guidelines.

**Results:**

The number of published papers on sepsis and the microcirculation has increased steadily over the last 25 years. We identified 11 experimental animal studies comparing the effects of different fluid resuscitation regimens on the microcirculation. Heterogeneity precluded any meta-analysis.

**Conclusions:**

Few animal model studies have been published comparing the microcirculatory effects of different types of fluid resuscitation for sepsis and septic shock. Biologically relevant animal model studies remain necessary to enhance understanding regarding the mechanisms by which fluid resuscitation affects the microcirculation and to facilitate the transfer of basic science discoveries to clinical applications.

## Review

### Introduction

Sepsis is a syndrome induced by infection that is characterized by physiologic, pathologic, and biochemical abnormalities [1], thus causing a substantial primary disease burden and co-morbidity [2]. Treatment guidelines globally recommend correction of haemodynamic abnormalities via the rapid administration of fluid boluses and blood transfusion to restore macro-circulatory parameters such as cardiac output and blood pressure [3–9]. Whilst there is general consensus on the key pillars of sepsis management—such as early recognition, source control, and timely antibiotic administration—there are a number of controversial issues surrounding volume resuscitation (type of fluid, dose, and rate). Treatment guidelines focus upon the normalization of macro-circulatory perturbations, with less attention to restoring microcirculatory dysfunction, which can only be resuscitated in the early period and has been shown to occur early in the disease process [10] and to persist in the later stages of volume-resuscitated sepsis and septic shock [10–18].

The microcirculation is an elaborate network of blood vessels, comprised of arterioles, venules, and capillaries that are lined with a dynamic endothelial-glycocalyx layer [19]. This network together forms the largest organ system in the body [20]. Microcirculatory function of coupling the delivery of metabolic substrates to respiring tissues, relative to requirements, and the removal of metabolic products, has been shown to be the main prerequisite for adequate tissue oxygenation and organ function [17]. Sepsis causes endothelial activation and the breakdown and shedding of the glycocalyx, leading to microcirculatory dysfunction. Progressive microvascular and organ dysfunction in sepsis and septic shock have been described extensively in literature [16–18, 21–34], and impaired microcirculatory function has been shown to be an independent predictor of mortality [11, 26]. Whilst it has been postulated that resuscitation of sepsis by rapid fluid administration may worsen microcirculatory dysfunction, there have been reports of glycocalyx stabilization by fresh frozen plasma (FFP) [35]. Increased microcirculatory flow during resuscitation for sepsis has been associated with reduced organ failure at 24 h without substantial differences in global haemodynamics, supporting the hypothesis that targeting microcirculation distinct from the macro-circulation could potentially improve organ failure in sepsis [36] and hence have a prognostic role and be the target of therapeutic interventions [37].

Description of microcirculatory alterations in septic animal models has been done using several techniques such as intravital microscopy, laser speckle imaging, laser Doppler flowmetry [32] and videomicroscopic techniques, such as orthogonal polarization spectroscopy and sidestream dark-field imaging [38]. Intravital microscopy entails imaging live animals at microscopic resolution [39] via trans-illumination or epi-illumination and recording pictures by means of low-light level silicon-intensified or charge-coupled video cameras [40]. It enables the in vivo morphological viewing of the microcirculation, as well as the quantification of micro-haemodynamic functional capillary density (FCD), endothelial integrity and cellular interactions when used in combination with fluorescent markers [40]. Laser speckle is an interference pattern produced by light reflected or scattered from different parts of a laser-illuminated surface [41]. The motion of particles in the laser-illuminated medium causes spatial and temporal fluctuations, producing an interference pattern consisting of bright and dark areas (i.e., the so-called speckles) visualized on a detector [42]. Laser Doppler produces an indirect measure of flow by quantifying shift in the Doppler frequency of a monochromatic laser light signal (helium-neon laser, 632.8 nm) that is scattered by red blood cells moving through the microcirculation [41, 43]. Light which is backscattered from moving erythrocytes undergoes a shift in frequency that is proportional to their velocity in accordance to the Doppler principle [44], hence making it possible to obtain reproducible measurements of blood flow at a single spot defined by the incident and Doppler-shifted reflected light [45]. Sidestream dark-field (SDF) imaging technology is based on improved orthogonal polarized spectral (OPS) imaging, whereby concentrically placed light-emitting diodes (LEDs) provide pulsed synchronous illumination of the microcirculation at a central wavelength of 530 nm for optimal absorption by the haemoglobin in red blood cells, independent of the oxygenation state [38].

Volume resuscitation for treatment of septic shock has been the standard of care recommended by guidelines following evidence of reduction in persistent hypovolaemia and improved survival [7, 9, 46, 47]. These guidelines have been mainly based on observational studies and expert opinion, in the absence of supportive evidence from randomized controlled trials. Consequently, different types of fluid are used in resuscitation including crystalloids, colloids and blood transfusion.

Generally, there is a paucity of animal model studies on effects of fluid resuscitation on the microcirculation. A comparison of crystalloid and colloid in cecal ligation and puncture-induced sepsis in rodents concluded that crystalloid infusion produced better microcirculatory function as well as mortality benefit [48], with growing interest in comparison of fluid types [49, 50] and early versus late resuscitation in experimental animal models [51, 52]. Clinically, there is still controversy on the choice of resuscitation fluids. The trial on *Early Goal Directed Therapy* (EGDT) for treatment of sepsis and septic shock [47] was found to have no mortality benefit in subsequent trials [53–55]. However, lower mortality in septic patients resuscitated with albumin-containing solutions had been reported in a meta-analysis [56] but there was no significant mortality benefit of albumin compared to saline in the *Fluid Expansion as Supportive Therapy* (FEAST) randomized controlled trial [57]. A Cochrane meta-analysis comparing colloid to crystalloid fluid resuscitation, in a heterogeneous group of critically-ill patients, failed to demonstrate any tangible benefits on mortality [58]. Emerging research data highlights an urgent need to review volume resuscitation strategies and adjunct vasopressor use in sepsis and septic shock.

Mechanistic research is therefore essential to define cause and effect. However, whilst animal research is advocated for investigating mechanisms of disease process and testing therapeutic interventions, discordant comparisons of treatment effects between animal experiments and clinical trials have arisen, due to the failure of animal models to adequately mimic clinical disease [59].

The aim of this review was to identify clinically relevant experimental animal models that have been used to assess the microcirculatory effects of fluid resuscitation treatment for lipopolysaccharide (LPS)-induced sepsis. We therefore examined the evidence available on the effects of fluid resuscitation on the microcirculation in LPS-induced sepsis and septic shock.

### Methods

#### Search strategy

A systematic search was conducted in two indexed online databases—PubMed/MEDLINE and EMBASE—for articles describing the microcirculation in sepsis and septic shock, published from 1 January 1990, through 31 December 2015, in accordance with PRISMA guidelines [60]. In PubMed/MEDLINE, the search terms used in [MeSH Terms] or [All Fields] were ‘sepsis’ OR ‘septicaemia’ OR ‘septic shock’ AND ‘microcirculation’. In EMBASE, the keywords used were ‘(microcirculation AND sepsis)’, ‘(microcirculation AND septicaemia)’, and ‘(microcirculation AND septic shock)’.

#### Selection criteria

All abstracts identified through the searches were compiled in Endnote® (Thomson Reuters) and screened for relevance, after removing duplicates. Publications were eligible for inclusion in the review if they were conducted on animal models of LPS-induced sepsis and septic shock and compared different types of fluid resuscitation, with or without a control group. Full published manuscripts of studies considered relevant were retrieved and reviewed. Cited publications within the retrieved articles were also screened for relevance. We restricted our review to studies assessing the effects of fluid resuscitation on the microcirculation in sepsis/septic shock and articles either written in English or translated into English.

### Results

**Fig. 1:**
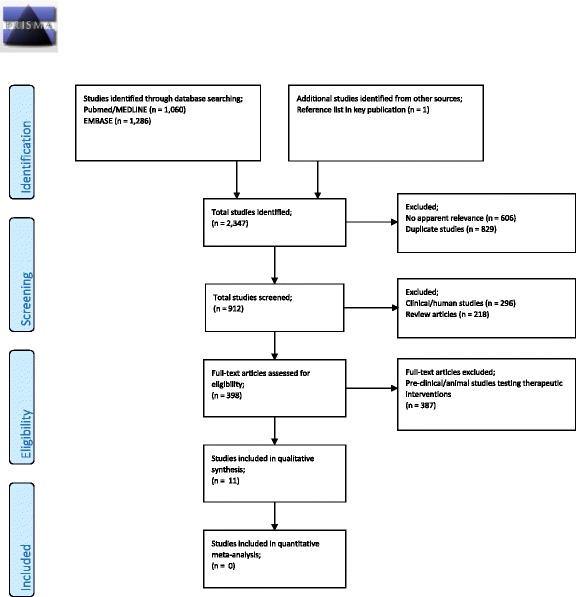
PRISMA flow diagram for experimental animal models of microcirculatory fluid resuscitation in septic shock

**Fig. 2:**
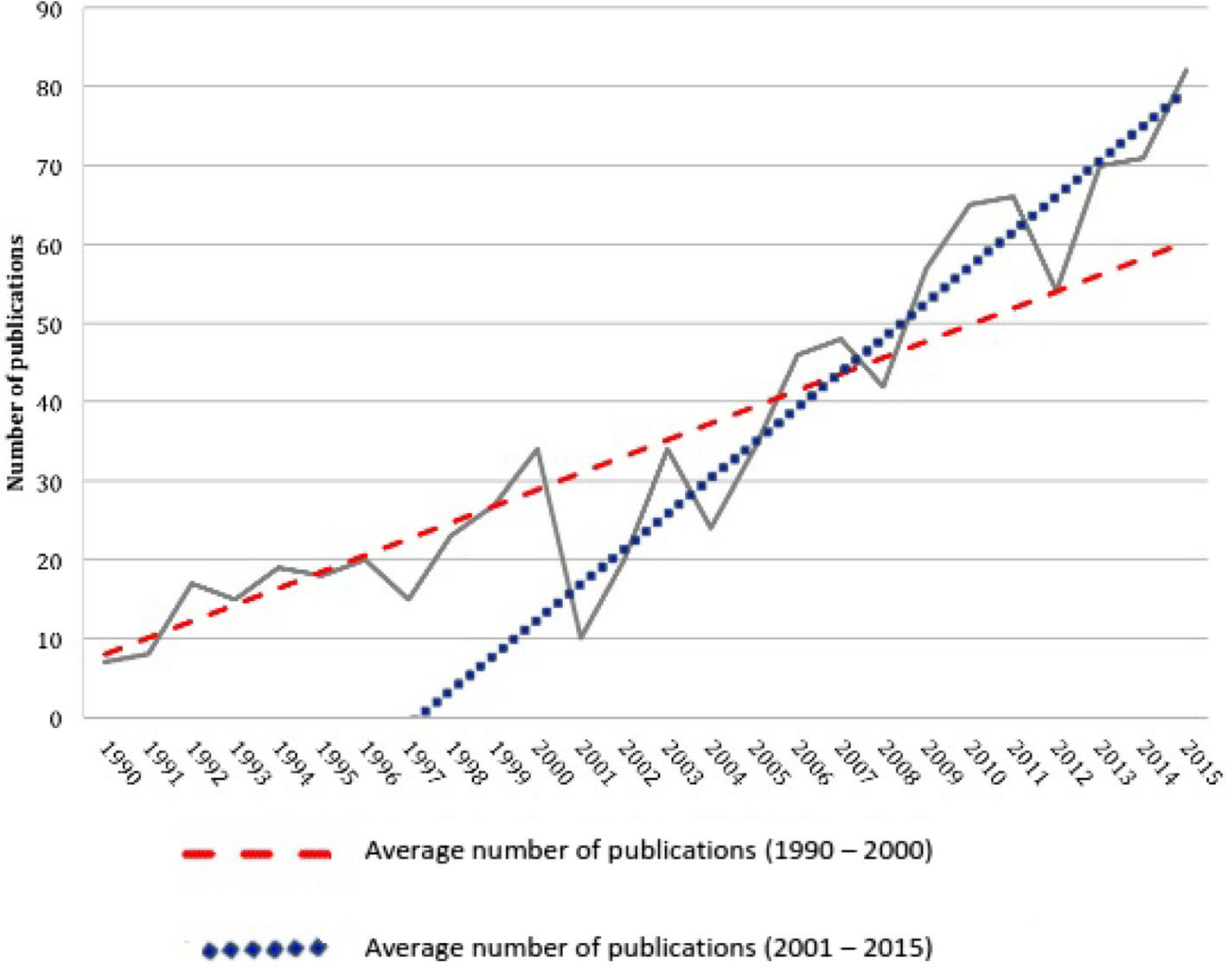
Number of unique publications on the microcirculation in sepsis and septic shock retrieved by year of publication from 1 January, 1990 to 31 December, 2015

A total of 2346 articles were retrieved from our search of published literature. Initial screening to remove duplicates and studies with no apparent relevance yielded 912 unique articles (Fig. 1). There has generally been a gradual increase in the number of published papers on sepsis and the microcirculation over the last 25 years (Fig. 2). After excluding non-experimental review articles, clinical studies and pre-clinical experiments investigating therapeutic interventions other than volume resuscitation, 11 studies were found to be relevant to the assessment of microcirculatory effects of volume resuscitation in animal models of LPS-induced sepsis. The methods used for quantification of microcirculation function, type and volume of resuscitation fluid administered and the resultant microcirculatory effects in the retrieved studies are presented in Table 1.

**Table 1:** Animal model studies assessing effects of volume resuscitation on the microcirculation in lipopolysaccharide-induced sepsis

Author	Publication year	Animal species	Number per arm	Methods used for microcirculation assessment	Type and mean volumes administered	Principal findings	Motivation for study
Maciel et al. [66]	1998	Dog	7	Quantification of oxygen extraction: Microcirculation was assessed indirectly by quantifying oxygen extraction in gas analyzers sampling to measure expired oxygen fraction and end-tidal carbon dioxide tension	a) 291 ± 62 mL (control group, isotonic saline 0.9%) b) 123 ± 12 mL (treatment group, hypertonic saline 7.5%)	Hypertonic saline resuscitation increases oxygen extraction compared to isotonic saline by improved microvascular perfusion	Assess whether a solution of hypertonic saline hydroxyl-ethyl starch can increase tissue oxygen extraction in endotoxic shock
de Carvalho et al. [61]	1999	Hamster	6	Intravital fluorescence microscopy: Microcirculation was assessed by intravital microscopy of cheek pouch tissue and counting extravasation sites of fluorescein isothiocyanate-labelled, FITC dextran	a) No sepsis control; 0.35 mL/100 g body weight for 4 min, 7.5% hypertonic saline b) and c) LPS groups 1 and 2 (no volume resuscitation controls) d) HS group; 0.35 mL/100 g body weight for 15 min prior to LPS e) HSD group; 0.35 mL/100 g body weight for 15 min prior and 4 min after the induction of LPS	Hypertonic saline with and without dextran reduce local and systemic endotoxin-inducedplasma leakage	Assess effect of hypertonic saline with and without dextran on endotoxin-induced vascular permeability in the cheek pouch microcirculation compared to systemically
Zhang et al. [67]	1999	Dog	7	Laser Doppler perfusion monitoring: Microcirculation was assessed by laser Doppler measurements obtained from ileum and liver microvasculature which were then used to calculate an arbitrary red blood cell flux index in 1 mm^3^ of tissue in each organ	a) No fluid resuscitation control group b) 20 mL/kg/h 0.9% normal saline	Microvascular depression in endotoxaemia was more severe in the liver than in the intestinal mucosa but increased similarly after initial resuscitation	Compare alterations in hepatic and intestinal mucosal microcirculation during the acute phase of blood flow reduction in endotoxic shock and the effect of fluid resuscitation
Oi et al. [64]	2000	Pig	(7, 8 and 9)	Laser Doppler flowmetry: Microcirculation was assessed by intestinal blood flow laser Doppler measurements expressed in arbitrary laser Doppler perfusion units, PU	a) No fluid resuscitation control group b) 4 mL/kg over 10-min (0.9% isotonic saline in 6% dextran 70, ISD) c) 4 mL/kg over 10-min (7.5% hypertonic saline in 6% dextran 70, HSD)	Hypertonic saline improved intestinal mucosal blood flow better than isotonic saline and no resuscitation	Compare effects of hypertonic saline, isotonic saline and no resuscitation in endotoxin shock
Hoffmann et al. [62]	2002	Hamster	(7, 6 and 8)	Intravital fluorescence microscopy: Microscopy was assessed by intravital microscopy on dorsal skin-fold chamber and computation of; (a) Functional capillary density, FCD (i.e. length of all erythrocyte-perfused nutritive capillaries per observation area) (b) Vascular permeability quantified by extravasation of fluorescein isothiocyanate-labelled, FITC dextran	a) No fluid resuscitation control group b) 16 mL/kg HES c) 66 mL/kg 0.9% isotonic saline	Synthetic hydroxyethyl starch (HES) preserved the functional capillary density (FCD) compared to saline and no resuscitation	Assess and compare effects of different volume support administered in endotoxin-induced microcirculatory disorders
Anning et al. [63]	2004	Rat	(5, 6 and 7)	Intravital fluorescence microscopy: Microcirculation was assessed by intravital microscopy on an exteriorised loop of intestine and its associated mesentry and computation of; (a) Measurements of the rolling velocity of all leucocytes entering a microvessel and leucocyte flux (i.e. the number of rolling leucocytes) were defined as adherent if stationary for >30 s (b) Vascular permeability quantified by extravasation of fluorescein isothiocyanate-labelled bovine serum albumin (FITC-BSA)	a) No fluid resuscitation control group b) 16 mL/kg/h (0.9% saline) c) 16 mL/kg/h (5% albumin)	Lipopolysaccharide-induced albumin flux, leucocyte rolling and adhesion in the microcirculation was reduced by both 0.9% saline and 5% human albumin solutions	Assess effect of fluid administration on lipopolysaccharide-induced changes in mesenteric microcirculation
Dubin et al. [68]	2008	Sheep	7	Sidestream dark-field imaging: Microcirculation was assessed by the following measurements obtained from sublingual mucosa and intestinal mucosa and serosa (three different regions within each site and each image was divided into four quadrants) (a) Microvascular flow index, MFI [i.e. based on the diameters, blood capillaries were classified as small (10–25 μm), medium (26–50 μm) or large (51–100 μm) and flow was scored as no flow (0), intermittent flow (1), sluggish flow (2), continuous flow (3) or hyperdynamic flow (4). MFI calculated as the sum of *each quadrant score divided by the number of quadrants in which the vessel type is visible*] (b) Percentage of perfused villi, PV% [i.e. the number of villi in each video were counted and semi-quantitatively classified as perfused, heterogeneously perfused or unperfused; PV% was calculated as number of perfused villi divided by the total number of villi]	6% HES^a^	Hydroxyethyl starch fluid resuscitation restored microcirculation in the sublingual and intestinal serosa but not in the intestinal mucosa	Test hypothesis that persistent villi hypoperfusion explains intramucosal acidosis after resuscitation for endotoxaemic shock
Legrand et al. [52]	2011	Rat	(5 and 7)	Laser speckle imaging: Microcirculation was assessed by the following measurements obtained from the renal cortex (a) Microvascular perfusion histograms based on laser speckle imaging perfusion maps (b) Microvascular oxygen tension histograms based on phosphorimetry	a) Early resuscitation group 40 mL/kg in 300 min (HES), administered as 20 mL/kg/h in the first hour and 5 mL/kg/h for the remaining duration of the protocol b) Late resuscitation group 30 mL/kg in 300 min (HES), administered as 20 mL/kg/h in the first hour and 5 mL/kg/h for the remaining duration of the protocol	Despite immediate hydroxyethyl starch fluid resuscitation being better than delayed resuscitation, overall prevention of renal macrovascular hypoperfusion did not fully prevent renal microcirculatory dysfunction	Test hypothesis that prevention of endotoxaemia-induced hypotension by immediate fluid resuscitation would prevent development of renal microcirculatory dysfunction
Andersson et al. [15]	2012	Sheep	(5 and 8)	Laser Doppler flowmetry and sidestream dark-field videomicroscopy: Microcirculation was assessed by the following measurements obtained from five sites in the ileal mucosa, with each site divided into 4 quadrants at each time-point. (a) Microvascular flow index, MFI [i.e. average flow of *all quadrants* scored as no flow (0), intermittent flow (1), sluggish flow (2) or continuous flow (3)] (b) Percentage of perfused villi, PV% [i.e. the number of villi in each video were counted and semi-quantitatively classified as perfused, heterogeneously perfused or unperfused; PV% was calculated as number of perfused villi divided by the total number of villi] (c) Heterogeneity index, HI [i.e. highest flow velocity minus lowest flow velocity divided by the mean MFI]	a) LPS group 519 ± (SD) 342 mL (HES)	Microcirculatory dysfunction persisted in fluid resuscitated endotoxaemic shock despite increased regional blood flow	Test hypothesis that in hyperdynamic endotoxaemic shock, intestinal microcirculatory dysfunction will be present despite increased regional blood flow
Duburcq et al. [65]	2014	Pig	5	Laser Doppler flowmetry: Microcirculation was assessed on the skin blood flow using laser Doppler measurements expressed in arbitrary perfusion units, PU [i.e. peak flow was defined as the highest flow signal obtained post-pneumatic occlusion of blood flow to the legs. Duration of the flow signal was also recorded]	a) 0.9% sodium chloride group 5 mL/kg/h b) 8.4% hypertonic sodium bicarbonate 5 mL/kg/h c) 11.2% hypertonic sodium lactate 5 mL/kg/h	Hypertonic sodium lactate solution improves microvascular reactivity with a negative fluid balance	Investigate effects of hypertonic sodium lactate compared to sodium chloride on the microcirculation in endotoxic shock
Lopez et al. [51]	2015	Pig	–	Sidestream dark-field videomicroscopy: Microcirculation was assessed by the following measurements obtained from the average of 12 quadrants (i.e. three videos of sublingual mucosa, four quadrants each); (a) Microvascular density, MVD [i.e. number of vessels per mm^2^ in sublingual mucosa] (b) Microvascular flow index, MFI [i.e. average flow of *individual vessels* scored as no flow (0), intermittent flow (1), sluggish flow (2) or continuous flow (3)] (c) Heterogeneity flow index, HFI [i.e. highest MFI minus lowest MFI divided by mean MFI] (d) Proportion of perfused vessels, PPV [i.e. total number of vessels minus number of vessels with flow = 0 or 1 divided by total number of vessels] (e) Perfused vessel density, PVD [i.e. MVD multiplied by PPV]	a) LPS group 8 mL/kg/h (saline) b) Early resuscitation protocol, ERP 250 mL/h for 2 h (Haemacell) c) Sham 8 mL/kg/h (saline)	Early resuscitation restored macro-haemodynamic parameters but microcirculatory alterations persisted	Assess systemic and microcirculatory correlation of early resuscitation for endotoxic shock

^a^Volume of resuscitation fluid administered not described

#### Study characteristics

The featured 11 studies involved both small rodent models, included two hamster studies (*n* = 58) [61, 62], and two rat studies (*n* = 57) [52, 63], and large mammal models, which included three pig (*n* = 64) [51, 64, 65], two dog (*n* = 28) [66, 67] and two sheep studies (*n* = 27) [15, 68]. In each instance, sepsis was induced by intravascular LPS infusion post induction of appropriate anaesthesia.

#### Microcirculatory assessment and outcome

In the small rodent models, microcirculatory function was assessed primarily by intravital fluorescence microscopy [61–63], with one study using laser speckle imaging [52]. Laser Doppler flowmetry and/or videomicroscopic sidestream dark-field (SDF) imaging were largely used for microcirculatory assessments in the larger mammalian models [15, 51, 65, 67]. One study utilized indirect quantification of oxygen extraction to assess microcirculatory function [66]. Six studies reported improved blood flow in the microcirculation and reduced extravasation of plasma following fluid resuscitation [61–64, 66, 67]. One study reported improvement of microvascular blood flow and oxygenation with overall negative fluid balance in sepsis [65], whilst three studies documented persistent microcirculatory dysfunction with volume resuscitation [15, 51, 52]. One study exhibited improved sublingual and serosal intestinal microcirculation, but persistent dysfunction in the intestinal mucosal villi within the same sepsis model resuscitated with hydroxyethyl starch (HES) [68]. None of the studies presented in this review used blood transfusion for volume resuscitation. Differences in the animal species, size of the study arms, methods of microcirculatory assessment, types and volume of fluid resuscitation administered and the experimental time-points for the induction of sepsis and resuscitation precluded any quantitative meta-analysis.

### Discussion

Preclinical animal research is a necessary adjunct to clinical trials as we seek to improve our mechanistic understanding of the pathophysiology of sepsis and the consequent effects of volume resuscitation. Different outcomes in the animal studies presented in this review reflect a state of equipoise regarding the effectiveness of volume resuscitation on the microcirculation in the management of sepsis and septic shock.

The aetiology of microcirculatory dysfunction seen during sepsis is multi-factorial, including increased blood viscosity, reduced red blood cell deformability [69], neutrophil activation [70] and impaired vascular auto-regulation [20], leading to inadequate oxygen delivery to tissues. The mechanical properties of red blood cells are altered by effects of endotoxin binding directly to the red blood cells [71]. Increased vascular permeability occurs as a result of endothelial cell damage. Disruption of the endothelial-glycocalyx barrier exposes endothelial cell surface adhesion molecules that trigger the activation of mast cells, and the adhesion of platelets and white blood cells, thereby causing further inflammation [72] and nitric oxide production [73]. Nitric oxide, in turn, induces the relaxation of vascular smooth muscle, leading to increased blood flow shear stress and the production of pro-inflammatory cytokines, further worsening endothelial inflammation. Rapidly administered volume resuscitation therapy in the setting of a damaged endothelial-glycocalyx barrier alters cardiovascular haemodynamics [51] and may further exacerbate interstitial oedema directly by increasing intra-vascular hydrostatic pressure and indirectly through osmotic diffusion across the concentration gradient of leaked solutes.

Three of the studies presented in this review used intravital microscopy to assess the microcirculation, two demonstrating an improvement with fluid administration [61, 63] and one an improvement with hydroxyl-ethyl starch but not saline administration [62]. Only one study used laser speckle microcirculatory imaging [52] and showed no improvement following fluid administration. Three studies presented in this review used laser Doppler imaging and showed improved microcirculatory reactivity with fluid administration [64, 65, 67]. Microcirculatory assessment by sidestream dark-field imaging presented in this review exhibited heterogeneous effects in different tissues in one study [68], but no improvement in another [51]. Comparisons of different microcirculatory imaging techniques have been reported in literature [41, 74–76]. Investigators who conducted a comparative study on cochlear blood flow concluded that intravital microscopic measurements were more sensitive than laser Doppler measurements [77]. However, comparing the microcirculatory results attained with different methods is a significant challenge. We considered some of these, including videomicroscopic techniques, whilst offering direct visualization of the microcirculation, the tissue contact element is largely uncontrolled and may cause pressure-induced artefacts. On the other hand, laser techniques are susceptible to motion artefacts, so that combining techniques might enhance the accuracy of microcirculatory assessments [76]. Only one study presented in this review used a combination of techniques to assess the microcirculation (i.e., laser Doppler flowmetry and sidestream dark-field imaging) and reported persistent acidosis, microcirculatory and mitochondrial dysfunction despite resuscitation with hydroxyethyl starch (HES) fluid [15]. Routine microcirculation assessment is rarely performed owing to heterogeneity of microvascular beds in different organs and complexity of assessment techniques. There was significant heterogeneity in the type and volume of resuscitation fluids used in the studies presented in this review. Clinically, substantial controversy still surrounds the choice of fluid for resuscitation. The *Saline versus Albumin Fluid Evaluation* (SAFE) study in adults similarly failed to identify any significant difference in the sepsis sub-group of patients [78], despite a lower unadjusted relative risk of death for albumin versus saline compared with non-septic patients [79]. The *Crystalloid versus Hydroxyethyl Starch Trial* (CHEST) revealed an increased rate of renal replacement therapy and adverse events (pruritus, rash) in intensive care unit patients resuscitated using colloid (6% hydroxyethyl starch, HES) compared to crystalloid (0.9% saline), with no significant difference in the 90-day mortality between the two groups [80]. For paediatric sepsis, the evidence base for fluid resuscitation upon definitive choices of fluids for resuscitation in severe infection and shock remains very weak [81], with most studies providing low quality of evidence or focusing on malaria and dengue. The FEAST trial documented a 45% relative (95% confidence interval, CI 13–86%) increase in 48-h mortality compared to control, with no significant difference between the saline and albumin bolus arms [57]. A subsequent systematic review formally assessing the evidence for bolus fluid resuscitation included 13 studies which met the inclusion criteria (4 general shock, 4 malaria, 4 dengue and 1 severe malnutrition). None were conducted in high-income countries and the only study to include a control arm (i.e. no fluid bolus arm; FEAST trial), found at 48 h a lower mortality in controls compared to those receiving saline or colloid boluses (relative risk for sepsis 0.69, 95% CI 0.54–0.89 and for malaria 0.64, 95% CI 0.46–0.91, respectively) [82]. Similarly, other studies have reported higher rates of morbidity and mortality with positive fluid balance in septic shock [83–89] and one retrospective pilot study reported good prognosis in patients who achieved a negative fluid balance within the first 3 days of septic shock [90]. Previous research showing transient pulmonary arterial hypertension that was induced by transfusion with donor blood, which had been stored for 35–42 days, is suggestive of a two-hit hypothesis that can be extrapolated to sepsis (first-hit) and volume resuscitation (second-hit) [91].

The pathogenesis of sepsis is complex, and evidence of higher rates of mortality with volume resuscitation therapy has led to the development of a two-hit hypothesis, highlighting the need for further mechanistic studies. Similarly, the heterogeneity in monitoring targets to guide resuscitation in septic shock has been recently highlighted [53–55]. Novel approaches and consensus in monitoring targets are required to preserve and evaluate the microcirculation in order to improve the treatment of sepsis.

## Conclusions

Microcirculatory resuscitation is an important therapeutic goal, as restoration of the macro-circulation alone fails to improve microvascular function. Transferring wet-bench discoveries to the clinical setting requires biologically relevant animal model studies to understand both the mechanisms by which fluid resuscitation affects the microcirculation and the role of the endothelial-glycocalyx in sepsis. One important priority for mechanistic research in sepsis is to compare the microcirculatory indices and outcomes achieved with different resuscitation strategies. Further research with defined therapeutic end-points and standardized fluid and microcirculatory assessment protocol is required as it remains unclear whether the heterogeneity that was seen was a result of the model used, resuscitation fluids used or the microcirculatory assessment techniques.

## Abbreviations

BSA: Bovine serum albumin
CHEST: Crystalloid Versus Hydroxyethyl Starch Trial
CI: Confidence interval
FCD: Functional capillary density
FEAST: Fluid Expansion as Supportive Therapy trial
FFP: Fresh frozen plasma
FITC: Fluorescein isothiocyanate
HES: Hydroxyethyl starch
HFI/HI: Heterogeneity flow index/heterogeneity index
LED: Light-emitting diode
LPS: Lipopolysaccharide
MeSH: Medical Subject Headings
MFI: Microvascular flow index
MVD: Microvascular density
OPS: Orthogonal polarized spectroscopy
PPV: Proportion of perfused vessels
PRISMA: Preferred Reporting Items for Systematic Reviews and Meta-Analyses
PU: Perfusion units
PV: Perfused villi
PVD: Perfused vessel density
SAFE: Saline versus Albumin Fluid Evaluation study
SD: Standard deviation
SDF: Sidestream dark-field imaging
